# Supplementary material for: Macrophage-derived IL-27 sustains autoreactive CD8+ cytotoxic T cells in autoimmune hepatitis
Source: bioRxiv. 2026 Sep 28:2026.09.25.754422. Preprint. [Version 1] doi: 10.64898/2026.09.25.754422 (PMC13648483; doi:10.64898/2026.09.25.754422)
Supplement: Supplementary file 1 [file NIHPP2026.09.25.754422v1-supplement-1.pdf]

**Supplemental Figure 1.**

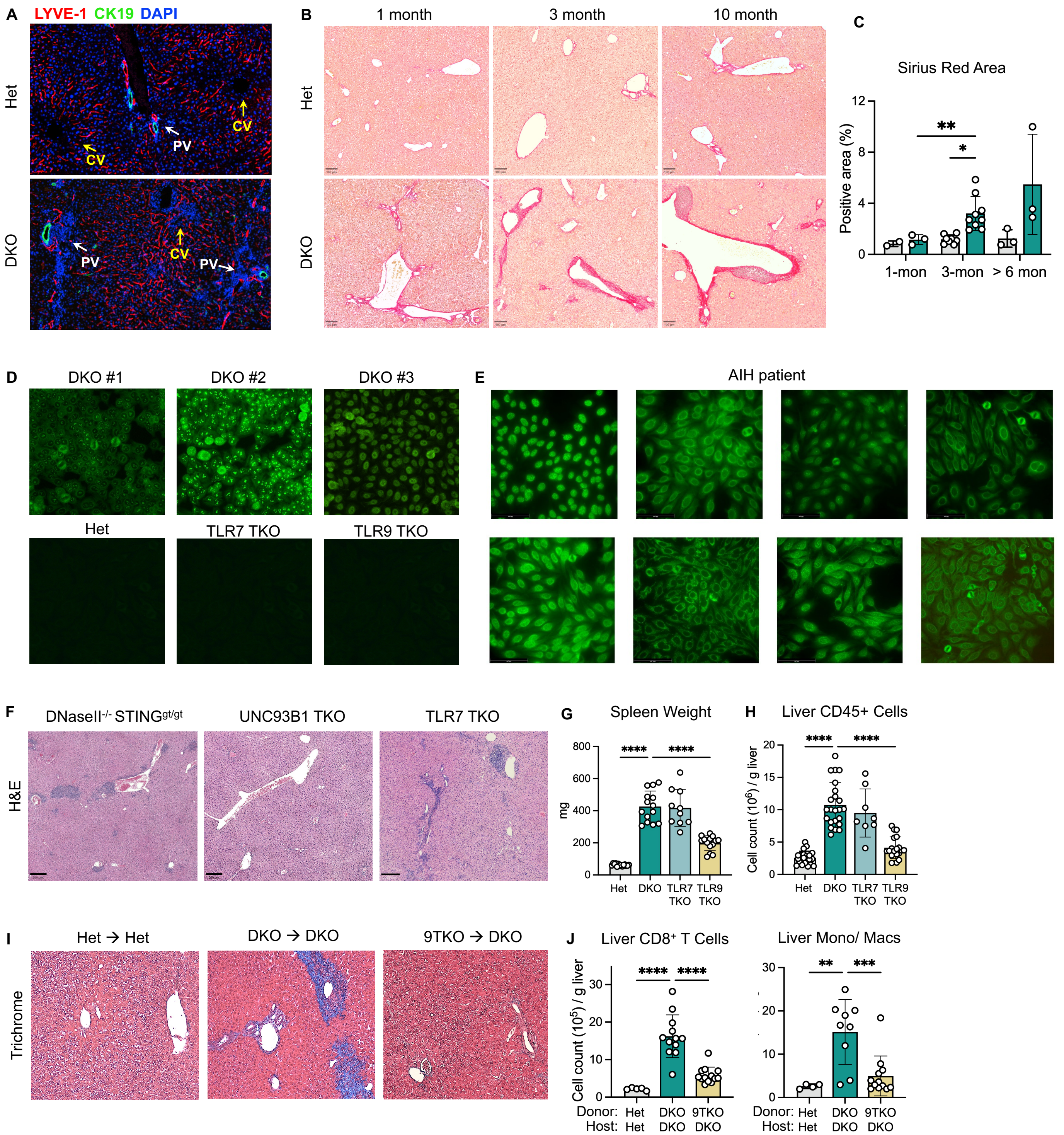


**Supplemental Figure 1.**

(A) Representative immunofluorescence images of liver section from Het, DKO, TLR7 TKO, and TLR9 TKO mice showing bile duct (CK19$^+$ green), sinusoidal endothelial cells (LVYE-1$^+$, red), and cell nucleus (DAPI, blue).
(B) Representative Sirius red staining (scale bar, 100um) of liver sections from Het and DKO mice at indicated age.
(C) Quantification of Sirius red positive liver area.
(D) Representative images of HEp-2 cells stained with sera from 3-4-month-old mice to reveal ANAs.
(E) Representative images of HEp-2 cells stained with sera from AIH patients to reveal different ANA staining patterns (n = 8).
(F) Representative H&E staining of liver sections from UNC93B1 TKO, DNaseII$^{-/-}$ STING$^{gt/gt}$ mice, and TLR7 TKO mice.
(G) Spleen weight of 3-4 month-old mice of indicated genotype (n = 10-14 per group).
(H) Absolute numbers of CD45$^+$ immune cells in the livers of 3-4 month-old mice (n = 7-25 per group).
(I) Representative Masson's trichrome staining (magnification,10X) of liver sections from chimeras 8-week post bone marrow transplant.
(J) Absolute numbers of indicated immune population in the livers of chimeras 8-week post bone marrow transplant (n = 9-14 per group)..

Data are pooled from at least 3 independent experiments and represented as mean ± SEM. *p < 0.05, **p < 0.01, ***p < 0.001, and ****p < 0.0001

**Supplemental Figure 2.**

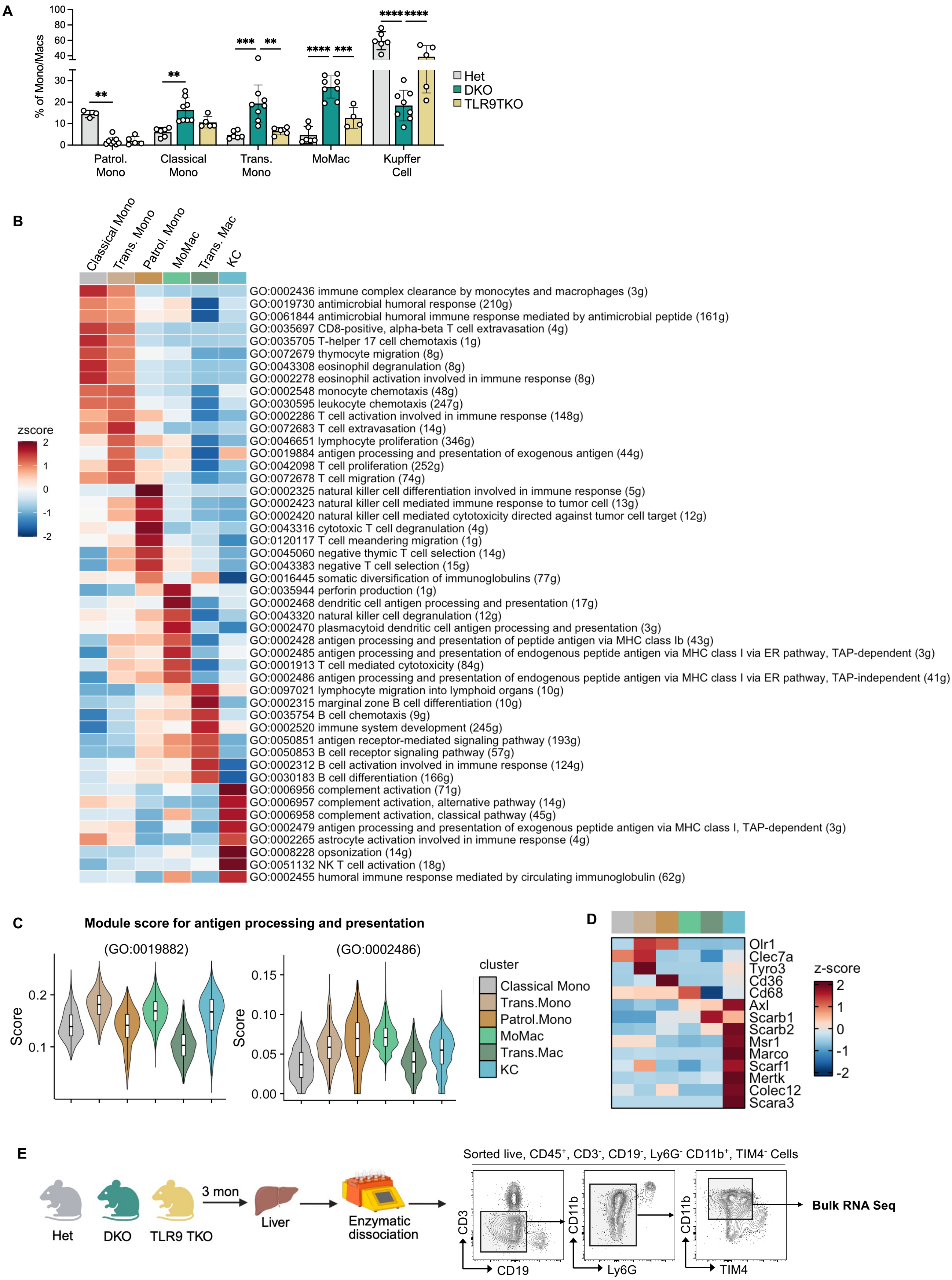


**Supplemental Figure 2.**

(A) Frequencies of indicated monocyte/macrophage subsets identified in figure 2D in Het, DKO, and TLR9 TKO livers (n = 5-8 per group). Data is pooled from 2 independent experiments and represented as mean ± SEM. *p < 0.05, **p < 0.01, ***p < 0.001, and ****p < 0.0001

(B) Heatmap showing enriched gene ontology (GO) terms in each monocyte/macrophage subset.

(C) Antigen-presentation signature scores across monocyte and macrophage subsets based on genes in GO pathways: antigen processing and presentation of endogenous peptide antigen via MHC class I via ER pathway, TAP-independent (GO:0002486), and antigen processing and presentation (GO:0019882).

(D) Heatmap showing RNA expression of scavenger receptor genes in indicated subsets across monocyte/macrophage subsets.

(E) Gating strategy used to sort monocyte-lineage cells for bulk RNA sequencing in figure 3I.

**Supplemental Figure 3.**

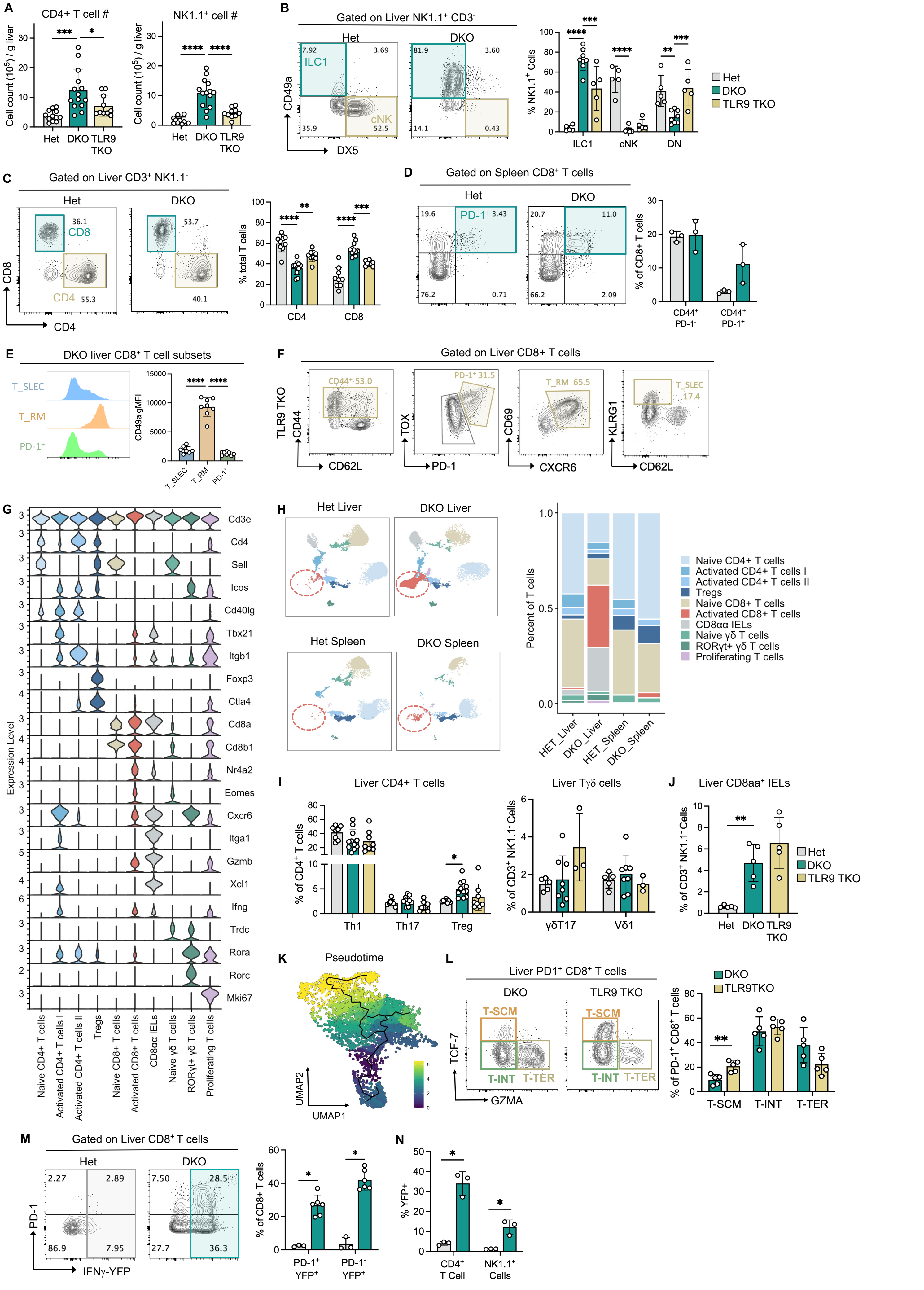

**Supplemental Figure 3.**

(A) Absolute number of $CD4^+$ T cells, and $NK1.1^+$ cells from livers of Het, DKO, and TLR9 TKO mice.
(B) Representative flow cytometry plots and frequencies of ILC1, conventional NK (cNK), and DX5 CD49a double negative (DN) cells among liver $CD3^-$ $NK1.1^+$ cells ( n=5-8 per group).
(C) Representative flow cytometry plots and frequencies of CD4+ and CD8+ T cells among liver $CD3^+$ $NK1.1^-$ cells (n = 9-14 per group).
(D) Representative flow cytometry plots and frequencies of $CD44^+$ $PD\text{-}1^+$, and $CD44^+$ $PD\text{-}1^-$ T cells among spleen $CD8^+$ T cells (n = 3 per group).
(E) Representative histograms and quantification of CD49a expression in the indicated DKO liver CD8+ T cell subsets (n = 8). Expression is reported as gMFI.
(F) Representative flow cytometry plots showing CD8+ T cell subsets identified in figure 3B in livers of TLR9 TKO mice.
(G) RNA expression of genes defining the T cell subsets in figure 3C.
(H) UMAP plots and relative abundances of T cell subsets from the livers and spleens of Het and DKO mice.
(I) Frequencies of Th1 ($T\text{-}bet^+$), Th17 ($ROR\gamma T^+$), and regulatory T cells (Treg) ($FoxP3^+$) among liver CD4+ T cells ( n= 8-11 per group); frequencies of γδT17 cells ($ROR\gamma T^+$, $IL17^+$) and Vδ1 ($T\text{-}bet^+$) T cells among liver γδ T cells ( n= 3-8 per group).
(J) Frequencies of liver CD8αα+ intraepithelial lymphocytes (IELs) among $CD3^+$ $NK1.1^-$ cells ( n= 5 per group).
(K) Pseudotime trajectory analysis of activated liver $CD8^+$ T cells by monocle 3.
(L) Representative flow cytometry plots and frequencies of T_SCM, T_INT, and T_TER cells among liver $CD44^+$ $PD\text{-}1^+$ $CD8^+$ T cells from DKO and TLR9 TKO mice (n = 5 per group).
(M) Representative flow cytometry plots and frequencies $YFP^+$ $PD\text{-}1^+$ and $YFP^+$ $PD\text{-}1^-$ cells among liver $CD8^+$ T cells in IFNγ reporter (GREAT) Het and DKO mice (n = 3-6 per group).
(N) Frequencies of $YFP^+$ cells among liver CD4+ T cells and NK1.1+ cells in IFNγ reporter (GREAT) Het and DKO mice (n = 3 per group).

Data are pooled from at least 2 independent experiments and are presented as mean ± SEM. *p < 0.05, **p < 0.01, ***p < 0.001, and ****p < 0.0001.

**Supplemental Figure 4.**

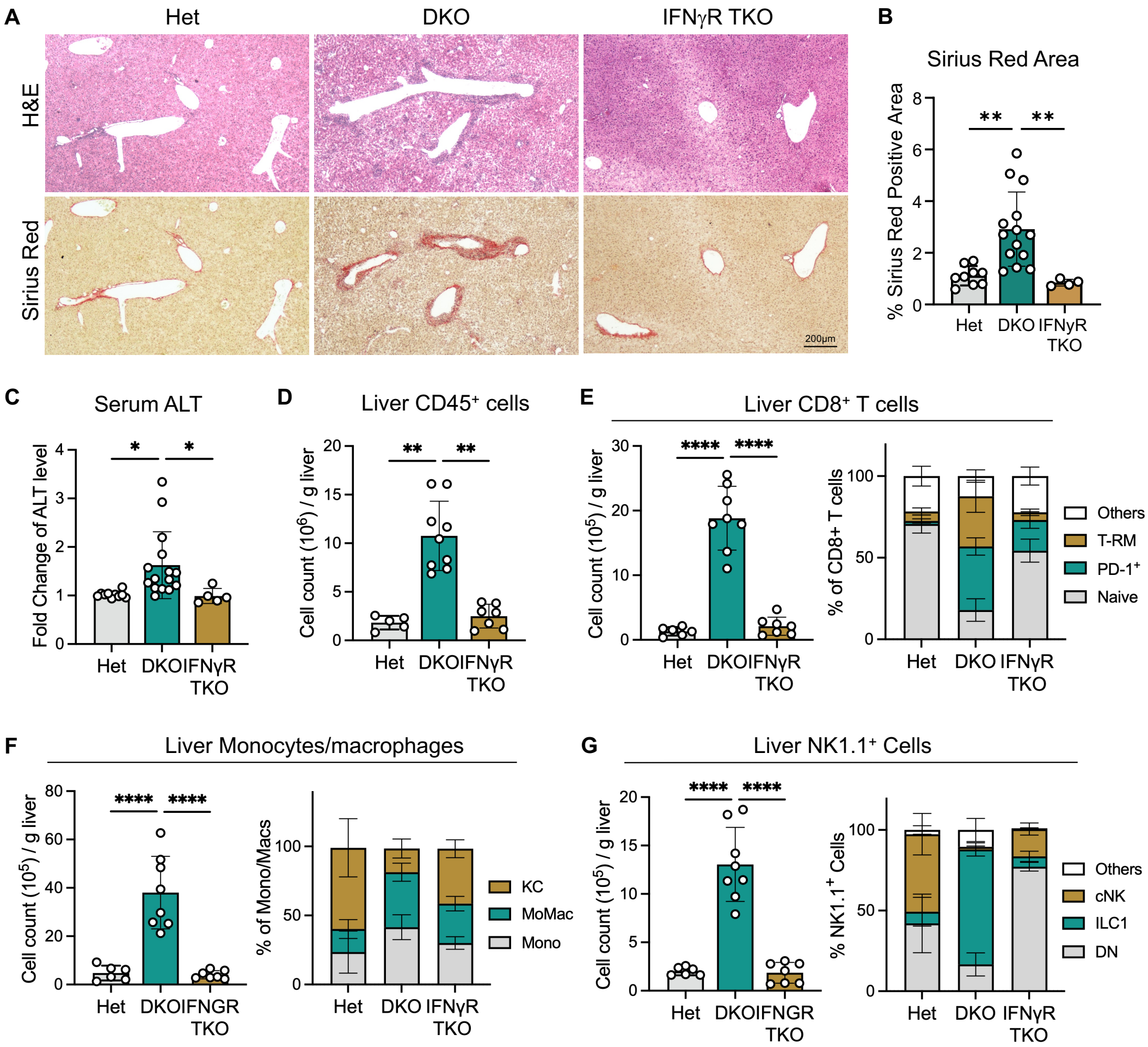


**Supplemental Figure 4.**

(A) Representative H&E, and Sirius Red staining (scale bars, 200um) of liver sections from 3-4-month-old mice.
(B) Quantification of Sirius red positive liver area (n = 4-14 per group).
(C) Serum ALT level relative to Het (n = 5-15 per group).
(D) Absolute numbers of $CD45^+$ immune cells in the livers (n = 5-9 per group).
(E) Absolute numbers and subset distribution of $CD8^+$ T cells in the livers (n = 6-8 per group).
(F) Absolute numbers and subset distribution of monocyte/macrophage populations in the livers (n = 6-8 per group).
(G) Absolute numbers and subset distribution of $NK1.1^+$ cells in the livers (n = 6-8 per group).

Data are pooled from at least 3 independent experiments and represented as mean ± SEM. *$p < 0.05$, **$p < 0.01$, ***$p < 0.001$, and ****$p < 0.0001$

**Supplemental Figure 5.**

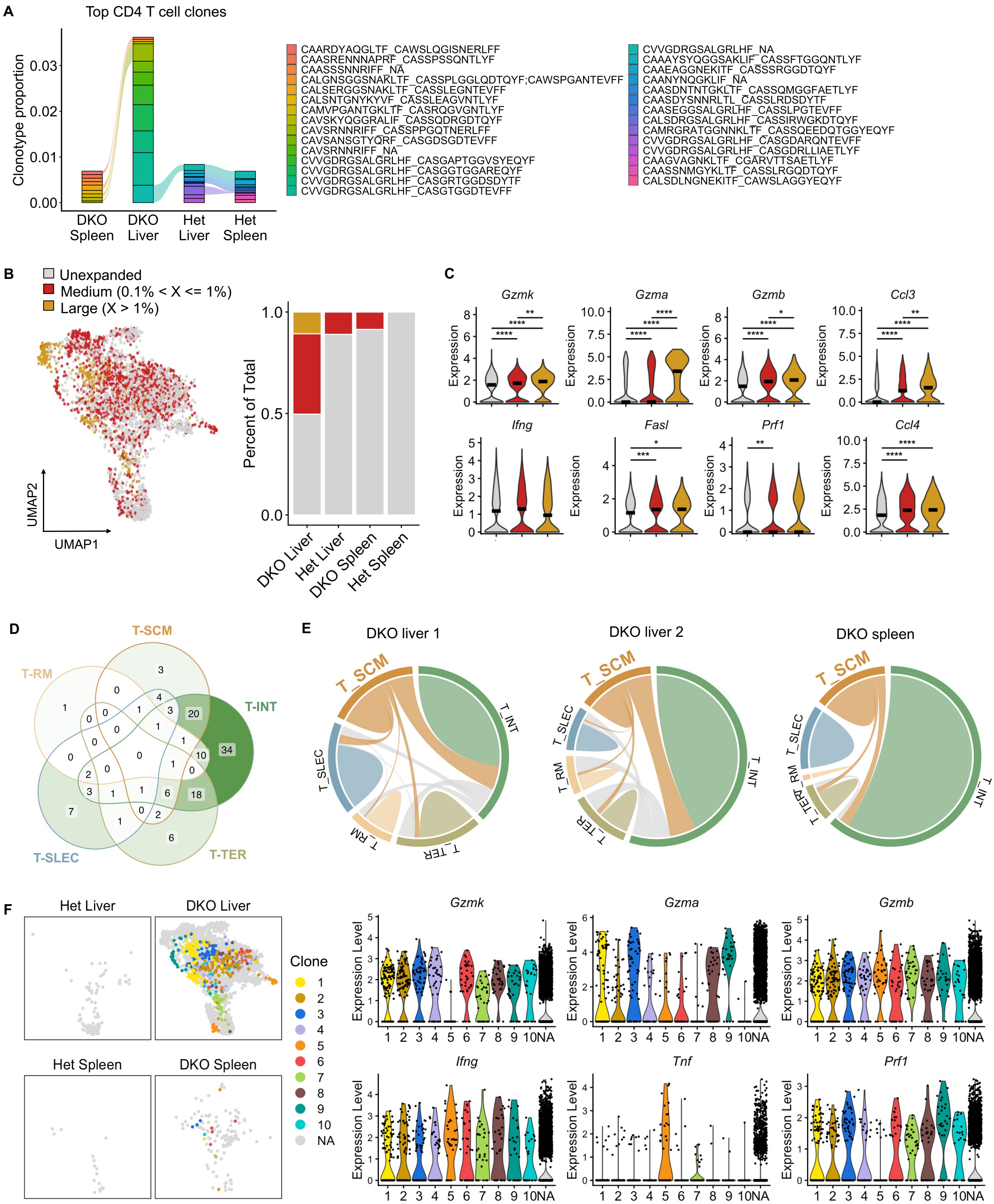


**Supplemental Figure 5.**

(A) Top expanded CD4⁺ T cell clonotypes across tissues, with each color representing one clone.
(B) UMAP plot of activated CD8⁺ T cells showing TCR clone size. Bar graph summarizes relative abundance of large (yellow), medium (red), and unexpanded (grey) clones within activated CD8⁺ T cells in the livers and spleens of Het and DKO mice.
(C) RNA expression of cytotoxic molecule across unexpanded, medium, and large TCR clones. *p < 0.05, **p < 0.01, ***p < 0.001, and ****p < 0.0001.
(D) Venn diagram showing the overlap of expanded TCR clonotypes (clone size > 0.1%) among indicated CD8+ T cell subsets. Numbers indicates clonotype counts within each unique or shared region.
(E) Chord diagrams showing shared TCR clonotypes among activated CD8+ T cell subsets in two individual DKO liver samples and pooled DKO spleen sample.
(F) UMAP plots of activated CD8⁺ T cells showing the tissue distribution of the top 10 shared TCR clonotypes identified in Figure 4I; and RNA expression of indicated cytotoxic molecules across the 10 TCR clonotypes.

**Supplemental Figure 6.**

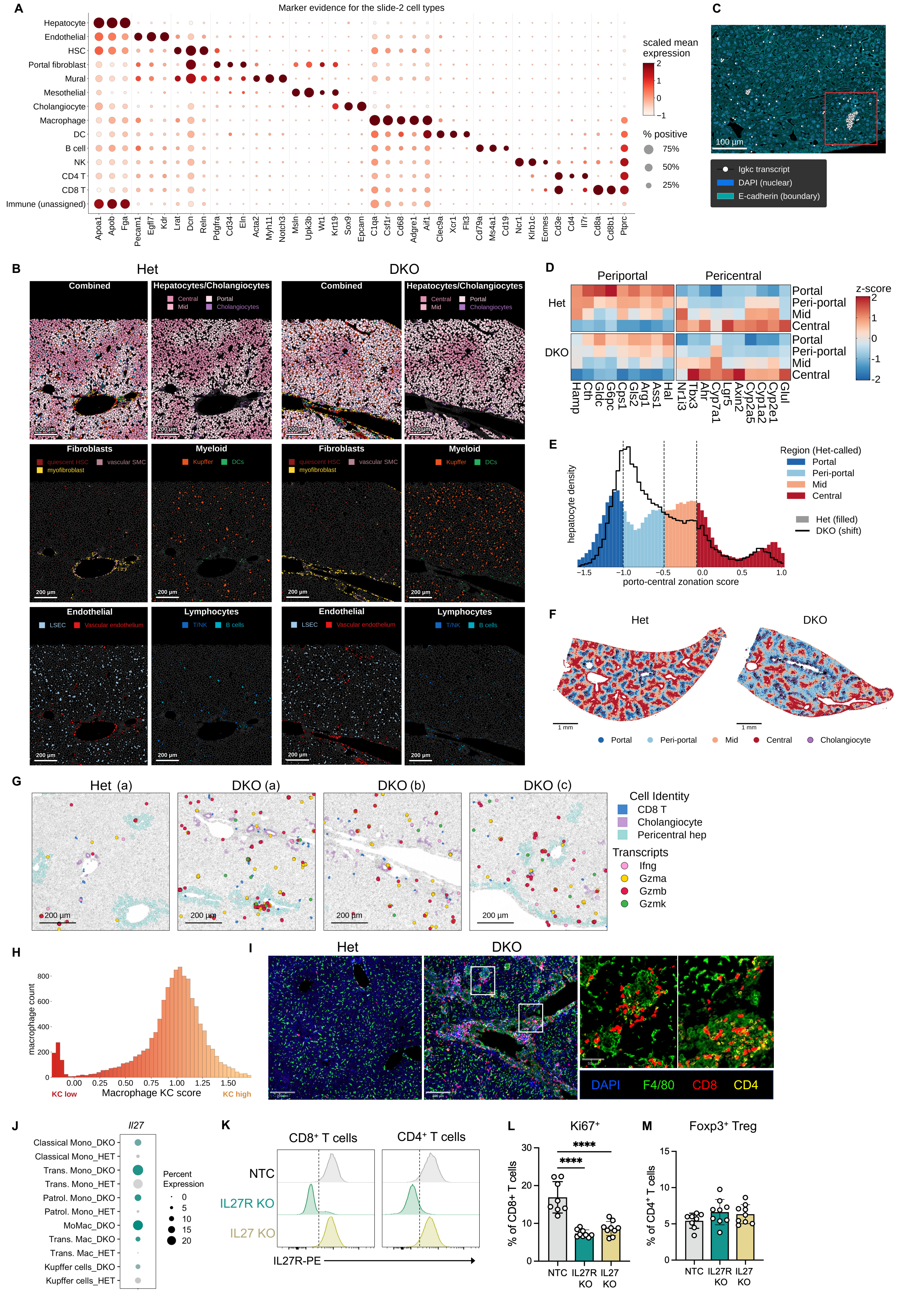

**Supplemental Figure 6.**

(A) Expression of genes that define clusters identified by Xenium spatial transcriptomics shown in figure 4A
(B) Representative spatial transcriptomic image of DKO liver section showing transcript distribution of Igkc, marker gene of plasma cells.
(C) Representative spatial transcriptomic images showing the distribution of the indicated parenchymal and non-parenchymal cell populations in Het and DKO liver sections.
(D) Heatmap showing expression of genes defining periportal and pericentral hepatocytes.
(E) Distribution of hepatocytes along the portal-to-central zonation axis in Het and DKO livers. Filled histograms show Het hepatocytes, and the black outline shows the distribution of DKO hepatocytes. Dashed lines indicate boundaries defining indicated regions based on the Het liver.
(F) Spatial mapping of the zonation pattern in Het and DKO liver sections (Scale bars, 1 mm).
(G) Representative spatial transcriptomic images showing the distribution of indicated T cell-associated transcripts in the same regions are same as shown in figure 4B.
(H) Distribution of macrophages according to continuous Kupffer cell (KC) score calculated based on mean expression of *Cd5l* and *Vsig4*.
(I) Representative immunofluorescence images (scale bar, 200um) of liver sections from Het and DKO mice showing macrophage (Green), CD8+ (red) and CD4+ (yellow) T cell clusters.
(J) Expression of Il27 across monocyte and macrophage subsets from Het and DKO livers identified by scRNA-seq.
(K) Representative histogram comparing surface IL27R expression on circulating CD8+ and CD4+ T cells in bone-marrow chimeras 9-week post transplantation.
(L) Frequencies of $Ki67^{+}$ $CD8^{+}$ T cells in the livers of bone-marrow chimeras 9-week post transplantation (n = 8=9 per group).
(M) Frequencies of Tregs in the livers of bone-marrow chimeras 9-week post transplantation.

Data shown in (K) – (M) are pooled from at least 2 independent experiments and represented as mean ± SEM. *p < 0.05, **p < 0.01, ***p < 0.001, and ****p < 0.0001

**Supplemental Figure 7.**

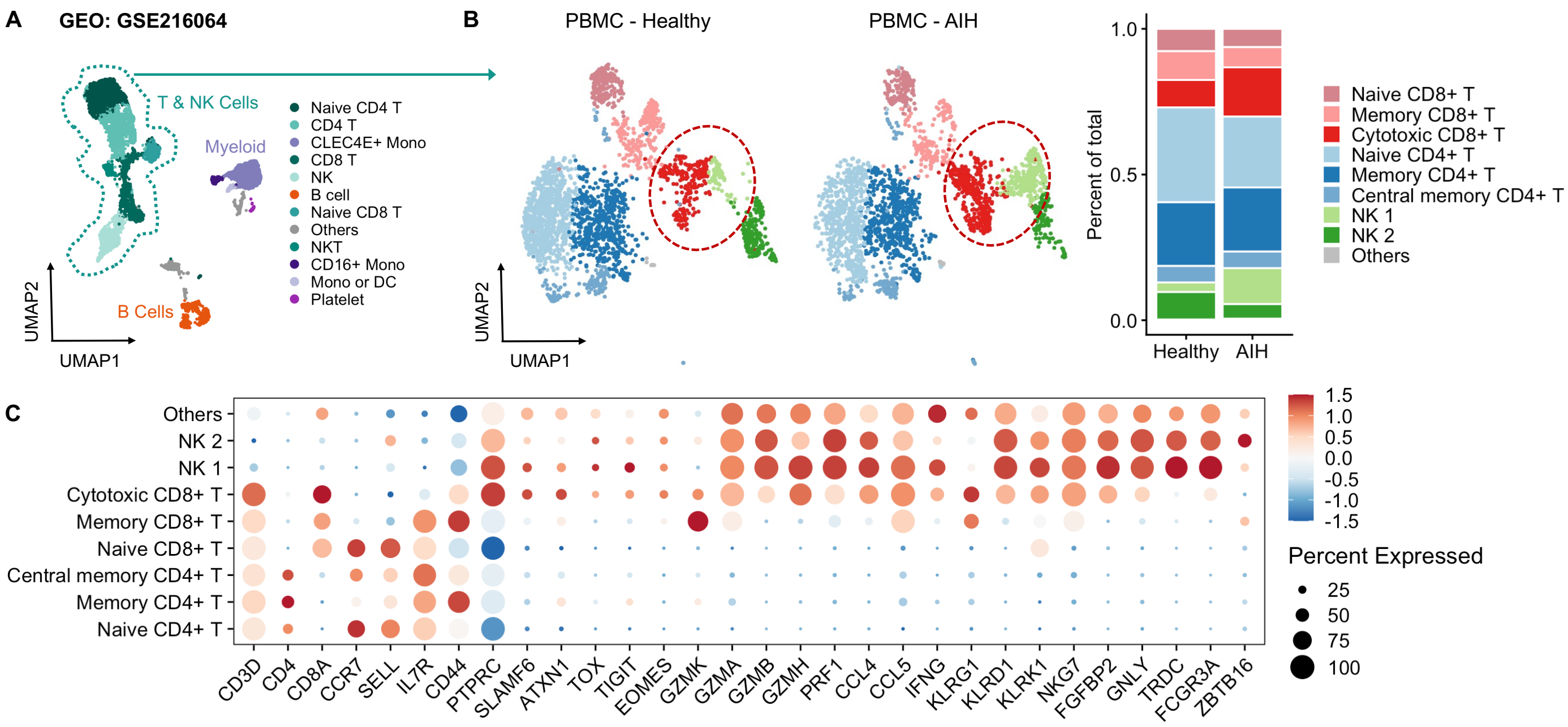


**Supplemental Figure 7.**

(A) UMAP plot of peripheral blood mononuclear cells (PBMCs) from healthy controls and patients with autoimmune hepatitis (AIH) in a published scRNA-seq dataset (GEO: GSE216064).
(B) UMAP plots and relative abundance of T and NK cell subsets in healthy controls and patients with AIH.
(C) Expression of cell-type-defining and cytotoxic effector genes across the T and NK cell subsets identified in (B).
